# A murine model for developmental dysplasia of the hip: ablation of CX3CR1 affects acetabular morphology and gait

**DOI:** 10.1186/s12967-017-1335-0

**Published:** 2017-11-10

**Authors:** George Feldman, Arlene Offemaria, Hind Sawan, Javad Parvizi, Theresa A. Freeman

**Affiliations:** 1 Division of Orthopaedic Research, Thomas Jefferson University, Philadelphia, USA; 2 Rothman Institute, Philadelphia, USA

**Keywords:** Developmental dysplasia of the hip, Acetabulum, Knock-out mouse, Gait analysis

## Abstract

**Background:**

Developmental dysplasia of the hip (DDH) is a debilitating condition whose distinguishing signs include incomplete formation of the acetabulum leading to dislocation of the femur, accelerated wear of the articular cartilage and joint laxity resulting in osteoarthritis. It is a complex disorder having environmental and genetic causes. Existing techniques fail to detect milder forms of DDH in newborns leading to hip osteoarthritis in young adults. A sensitive, specific and cost effective test would allow identification of newborns that could be non-invasively corrected by the use of a Pavlik harness. Previously, we identified a 2.5 MB candidate region on human chromosome 3 by using linkage analysis of a 4 generation, 72 member family. Whole exome sequencing of the DNA of 4 severely affected members revealed a single nucleotide polymorphism variant, rs3732378 co-inherited by all 11 affected family members. This variant causes a threonine to methionine amino acid change in the coding sequence of the CX3CR1 chemokine receptor and is predicted to be harmful to the function of the protein To gain further insight into the function of this mutation we examined the effect of CX3CR1 ablation on the architecture of the mouse acetabulum and on the murine gait.

**Methods:**

The hips of 5 and 8 weeks old wild type and CX3CR1 KO mice were analyzed using micro-CT to measure acetabular diameter and ten additional dimensional parameters. Eight week old mice were gait tested using an inclined treadmill with and without load and then underwent micro-CT analysis.

**Results:**

(1) KO mice showed larger a 5–17% larger diameter left acetabula than WT mice at both ages. (2) At 8 weeks the normalized area of space (i.e. size discrepancy) between the femur head and acetabulum is significantly larger [38% (p = 0.001)–21% (p = 0.037)] in the KO mice. (3) At 8 weeks gait analysis of these same mice shows several metrics that are consistent with impairment in the KO but not the WT mice. These deficits are often seen in mice and humans who develop hip OA.

**Conclusion:**

The effect of CX3CR1 deletion on murine acetabular development provides suggestive evidence of a susceptibility inducing role of the CX3CR1 gene on DDH.

**Electronic supplementary material:**

The online version of this article (10.1186/s12967-017-1335-0) contains supplementary material, which is available to authorized users.

## Background

Developmental dysplasia of the hip (DDH) is a debilitating condition distinguished by an incomplete formation of the acetabulum and proximal femur leading to dislocation of the femur in the newborn and premature arthritis in the young [1]. While DDH affects 1 in 1000 newborns in the United States, there are well-defined pockets of high prevalence in Japan, Italy and other Mediterranean countries [2]. Because of its high prevalence and morbidity, screening programs involving ultrasound imaging of the hip in infants or manipulation of the femur are common place in most countries [3]. This methodology is reasonably accurate for detecting gross forms of hip dysplasia, but fails to identify the milder forms of this condition. It is this undetected sub-fraction of DDH patients that leads to early hip osteoarthritis in young adults causing over 40% of cases in the 20–40 years old age group [4]. A sensitive, specific and cost effective test has remained an elusive goal in orthopaedic medicine. If such a test were developed to identify newborns with this condition a device that immobilizes the femur during a crucial period of infancy called a Pavlik harness could be used to allow the acetabular labrum to fully form and obviate the debilitating sequelae of DDH.

Developmental dysplasia of the hip is a complex disorder having both an environmental and a strong genetic component, as shown by a high concordance between identical twins and high inheritance among closely related family members [5–9]. Previously, as a first step towards developing such a diagnostic test, a four generation, 72 member family that showed transmission of DDH was identified [10]. Linkage analysis revealed a 2.5 MB candidate region on chromosome 3 (LOD = 3.31). Whole exome sequencing of four affected family members uncovered a known SNP variant co-inherited by all 11 affected family members. This variant, rs3732378, causes a threonine to methionine amino acid change in the coding sequence of the CX3CR1 chemokine receptor. This mutation is predicted to damage protein function and increase susceptibility to a number of disorders in other organ systems [10]. It has a minor allele frequency in the Caucasian population of 0.145. Recently in a case–control study of 689 affected patients, and an equal number of controls, this chemokine variant was found to increase the risk of hip dislocation by a factor of 2.25 (OR = 2.25, 95% CI 1.42–3.56) after adjustment for gender [11]. To gain further insight into the potential function of this mutation we examined the effect of CX3CR1 ablation on the architecture of the mouse acetabulum and on the murine gait.

## Methods

### Mouse models

This research was conducted under guidelines established by the Animal Care and Use Committee (IACUC) at Thomas Jefferson University.

Ten 35 days old females (5 KO/5WT) from Taconic (C57BL/6NTac-[KO]CX3CR1 mouse line, Line #4167) and 16/56 days old (8KO/8WT) female mice were obtained from Jackson laboratory (B6.129P-Cx3cr1tm1Litt/J Stock No: 005582. In the exonic DNA of the Cx3cr1tm1Litt/J mice GFP is inserted resulting in the expression of GFP wherever this chemokine receptor would have been expressed).All experiments on mice were performed under conditions that conformed to standards set by TJU’s IACUC guidelines.

### MicroCT

WT mice were strain, sex and age matched with the transgenic mice and the carcasses were fixed and evaluated by microCT using Image-Pro Plus and Autovisualizer software (Media Cybernetics, Silver Spring, MD) to measure 11 parameters [area, major and minor axis, maximum, mean, minimum diameter, maximum radius, radius ratio, size (length) size (width)] for each slice and the space between the femur head and the acetabulum. The area of the space between the femur head and acetabulum for each slice was normalized.

### Gait analysis

DigiGait analysis was performed on 8 weeks old mice (the same ones on which microCT analysis was later performed) by Mouse Specifics, Inc (Boston) as previously described [12]. Briefly, each mouse was placed on a motor-driven treadmill with a transparent treadmill belt and imaged from beneath with a high-speed digital video camera. A minimum of 3 s of movie is required for Digigait analysis. Color images were converted to their binary matrix equivalents and the areas of the moving paws relative to the belt and camera were calculated throughout each stride. This was used to generate a dynamic gait signal of the paw placement relative to the treadmill belt and camera. Studies were conducted at a speed of 24 cm/s at an incline of 12° with and without a sled (attached to the mouse’s tail and equal to the individual mouse’s weight).

### Statistics

#### MicroCT analysis

A two tail t-test was conducted for all KO and WT samples to calculate the p value for every slice. Significant differences of the normalized area of the space between the femur head and the acetabulum are reported. Gait analysis: statistical analyses were carried out using GraphPad Prism 5.01 for Windows (GraphPad Software, San Diego) statistical software. One-way t-tests to analyse effects of a single variable, (i.e. genotype on gait) were performed.

Confidence intervals for both 5 and 8 week old mice were calculated (see Additional file 1: Figures S1 and Additional file 3: Figures S3).

#### Histology

Histologic analysis of the pelvises after they were demineralized, paraffin embedded, sectioned and stained with H and E.

#### Immunohistochemistry

Tissue slides were deparaffinized, rehydrated, and placed in antigen unmasking solution (Vector; Burlingame, CA), then washed and permeabilized with 0.5% Triton. Slides were blocked in 4% Bovine Serum Albumin (BSA, Equitech-Bio, Kerrville, TX) with 0.1% Tween 20 in PBS for 1 h. Primary antibodies were diluted 1:50 with 1% BSA with 0.1% Tween 20 in PBS, placed on the slides and incubated overnight at 4 °C. The following primary antibodies were used: GFP Tag polyclonal, Alexa Fluor 488, secondary fluorescent antibodies, applied for 1 h were, Alexa Fluor 488 donkey or Alexa Fluor 594 goat anti-rabbit (Invitrogen, Eugene, OR), then washed in PBS and coverslipped using Vectashield Hard Set with 4′,6-diamidino-2-phenylindole (DAPI, Vector, Burlingame, CA). A negative control sample was incubated with no primary antibody. All slides were imaged on an Eclipse E800 microscope (Nikon, Melville, NY) with an Evolution QEi Monochrome camera with LCD color filter (Q-imaging, Canada).

## Results

### MicroCT analysis

**Fig. 1:**
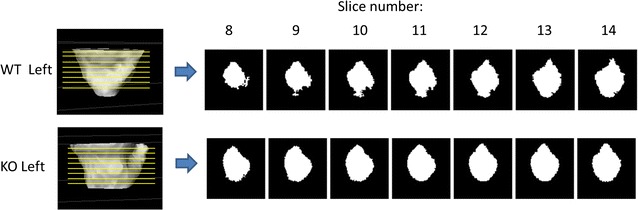
Micro-CT images of the Left Acetabulum of WT and KO 5 week old mice and 7 cross-sections (slices 8–14) proceeding medially

**Fig. 2:**
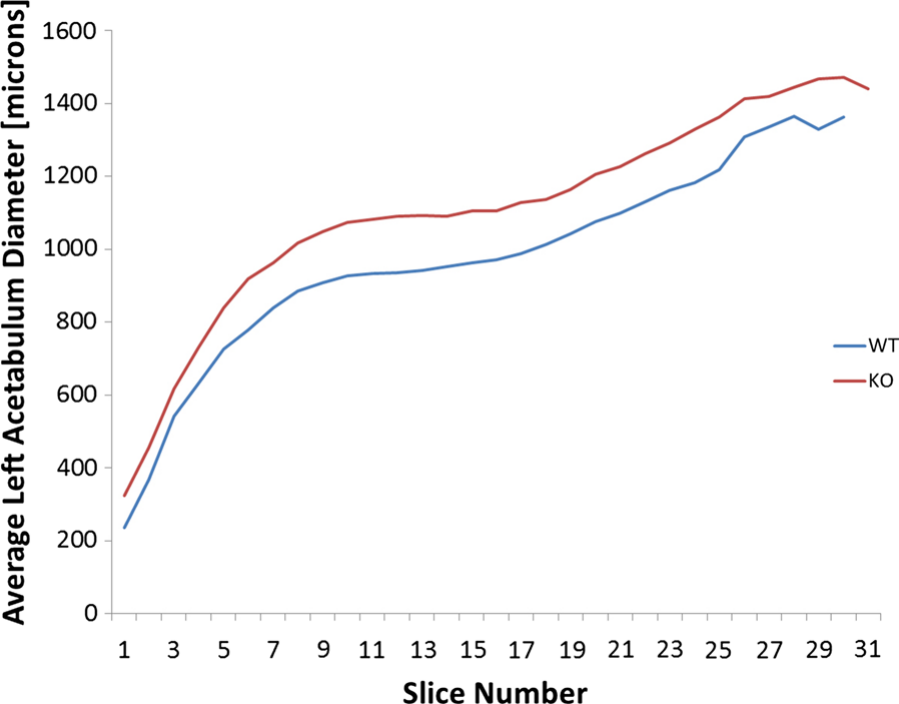
Average normalized diameter of the left acetabula of KO and WT 5 weeks old mice as a function of slice number proceeding medially. Average diameter differences in slices 8–17 were significant with p values ranging from 0.019 to 0.047

**Fig. 3:**
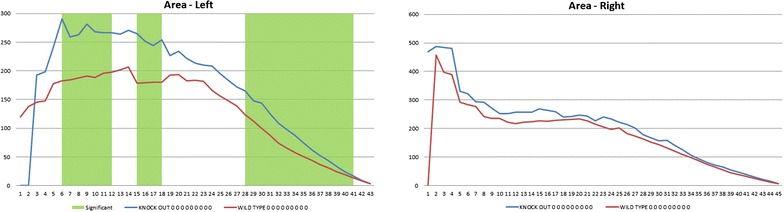
At 8 weeks normalized area of space between the femur head and left acetabulum ranging from 38% (p = 0.001) to 21% (p = 0.037). The right side showed no significant difference

The acetabula of 5 weeks old mice revealed that the left acetabulum of KO mice is 5.93% (p = 0.047) − 17.97% (p = 0.019) larger after normalization by femur head diameter. The size difference is significant over a range of tissue Sections (6–31) (Figs. 1, 2; Additional file 1: Figure S1, Additional file 2: Figure S2). These diameter differences continued to be seen at 8 weeks (Additional file 3: Figure S3). Depending on the cross-section the normalized area of space between the femur head and acetabulum at 8 weeks on the left side ranged from 21.5% (p = 0.037) to 45.4% (p = 0.012). No significant differences were found on the right side of either age (Fig. 3). Significant differences between KO and WT on the left side but not the right side also existed for the major and minor axes of the acetabulum, mean and minimum acetabular diameters, and the acetabular radius ratio (Additional file 3: Figure S3).

### Gait analysis

**Fig. 4:**
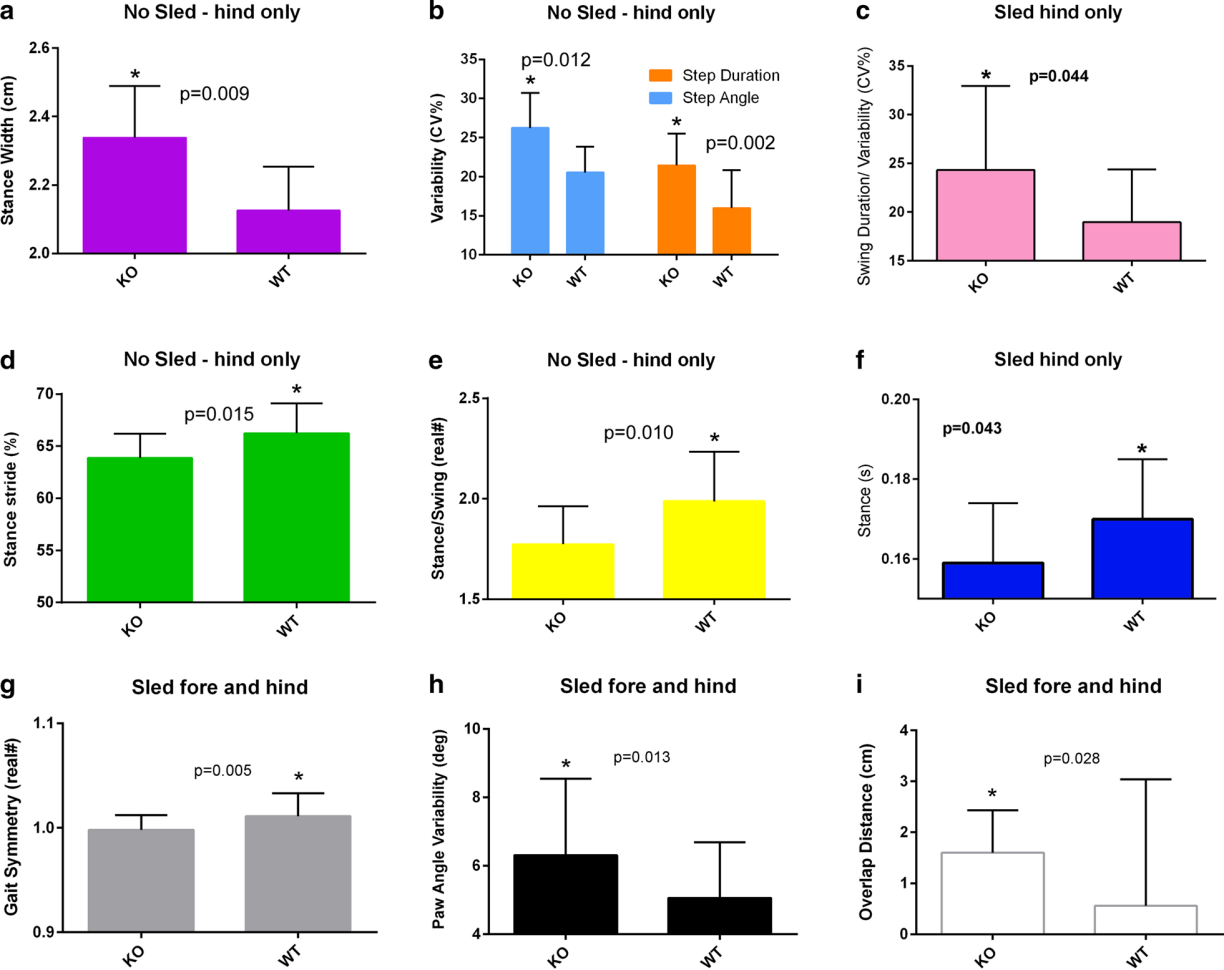
**a**–**i** Graphs comparing gait characteristics of KO and WT mice. Fore and hind limb gaits were measured separately. Studies were conducted at a speed of 24 cm/s at an incline of 12 degrees with and without a sled (attached to the mouse’s tail and equal to the individual mouse’s weight)

Stance width measured in cm is the perpendicular distance between the center points of either set of paws when each set of paws is in maximum contact with the treadmill surface. A larger stance width was observed in the KO mice with (p = 0.043) and without load (p = 0.009) (Fig. 4a). Step angle defined as the angle between left and right hind paws as a function of stride length and stance width. Step angle and step duration variability were observed to increase in the KO compared to WT (p = 0.012 and p = 0.002 respectively) (Fig. 4b). Swing duration variability is calculated by normalizing the variability (standard deviation) by dividing it by the mean. This variability was also seen in KO mice under load (p = 0.044) (Fig. 4c). Percent stance stride is the percent of the total stride duration that the paw is in any contact with the belt. Our results show a significant decrease in this measure (p = 0.015) (Fig. 4d). Stance/swing or the ratio of the stance phase time to swing phase time decreased in the KO mice (p = 0.010) (Fig. 4e). Stance time (in seconds) is the time duration of paw contact with the belt. Stance time decreased significantly under load (p = 0.043) (Fig. 4f). Gait symmetry, defined as the ratio of the forelimb stepping to hind limb stepping frequency, was significantly (p = 0.005) decreased in KO mice when they were pulling a load (Fig. 4g). Paw angle variability or the variation of the angle that the paw makes with the long axis of the direction of motion of the animal was found to significantly increase in the KO mice (p = 0.013) (Fig. 4h). Finally the overlap distance between the fore and hind paws was significantly greater in the KO mice under load (Fig. 4i).

**Fig. 5:**
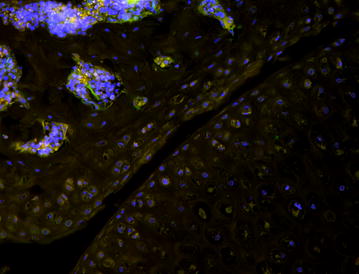
Photo micrograph of hip joint showing bone marrow cells and cartilage from CX3CR1 KO mouse expressing GFP where the chemokine receptor would have been expressed

Immuno-staining for GFP shows that CX3CR1 is expressed in the bone marrow cells of the hip joints of KO mice (Fig. 5). This observation is consistent with the expression pattern of this chemokine receptor [13].

To determine if there were any abnormalities in the bone density between the KO and WT, BV/TV of the hip acetabula was measured. The KO mice had a BV/TV which was 4.36% higher in the left hip (with a p value approaching significance p = 0.0769).

## Discussion

Evidence produced by the data outlined above provides insight into the mechanism by which the mutation found in the DNA of some patients with DDH could cause their disorder. We observed a persistence of the normalized unilateral acetabular diameter/femur head discrepancy at 5 and 8 weeks. The larger acetabulum relative to the femur head diameter at both 5 and 8 weeks in mice with the CX3CR1 gene ablated should produce greater joint laxity and instability—one of the cardinal signs of DDH. The sequelae of this joint laxity over time produces detectable gait abnormalities in humans. We observed these in our murine model and increases susceptibility to OA. Patients with DDH exhibit limping, toe walking, or a waddling, duck-like gait have easily dislocatable hips and develop early onset hip OA [14].

Gait analysis shows several metrics consistent with impairment. A larger stance width observed in the KO mice manifests itself in humans affected with OA as a postural adjustment for stability [15]. Swing duration variability increases such as that seen in the KO mouse are often seen in older human adults and individuals with osteoarthritis of the hip [16]. Similarly, gait symmetry was significantly decreased in the KO mice and in humans with hip OA as was the percent stance stride (the percent of total stride duration that the paw is in any contact with the belt) measure (p = 0.015) [17]. This gait metric is known to decrease in the mono arthritic rat model 4 h after intra-articular injection with carrageenan [18]. During normal gait in quadrupeds such as mice the forelimbs and hindlimbs show a stereotypical pattern of movement relative to one another. The hindlimbs are typically swung forwards such that hindpaws are plantar placed during stance, close to, but often a little ahead of, the position occupied by the forepaws during the preceding forepaw stance phase. The hindpaw stride therefore overlaps that of the forepaw stride in the direction of movement (antero-posterior axis). Injury or disease can lead to a change in the spatial relationship between forelimb and hindlimb strides such that the degree of overlap between the forepaw and subsequent hindpaw stance positions may increase or decrease [19].

In humans, DDH often manifests itself in a unilateral manner [9]. This one-sided expression was also observed in our murine model. The cause of this is unknown but is thought to be influenced by environmental or epigenetic factors such as the position of the fetus in the womb [8, 9]. To determine if there were any abnormalities in the bone density between the KO and WT, BV/TV of the hip acetabula was measured. The KO mice had a BV/TV which was 4.36% higher in the left hip (with a p value approaching significance p = 0.0769). This finding supports the observation of Hoshino and his colleagues, who noted increased bone volume in the CX3CR1 KO mouse. They found that in the absence of this chemokine receptor bone resorption was inhibited as compared to bone formation resulting in slightly higher bone density [20]. In addition to increased bone density, Hoshino and his colleagues found that CX3CR1 affected a number of aspects of bone metabolism in KO mice. CX3CR1–deficient bones showed elevated expression of Osterix/SP7, an essential osteoblast transcription factor. Additionally, they observed Osteocalcin a late marker to be down-regulated. Importantly, they found that CX3CR1-deficient pre-osteoblasts exhibited impaired differentiation [20]. Supported by our microCT and gait analysis of mice with an ablated CX3CR1 chemokine receptor and consistent with the finding of impaired differentiation, we hypothesize that this chemokine receptor may play a significant role in the progression of mesenchymal cell differentiation to chondrocytes or osteoblasts. Thus, a mutation in this chemokine receptor could delay maturation of the acetabulum and lead to a mild but significant change in morphology.

**Fig. 6:**
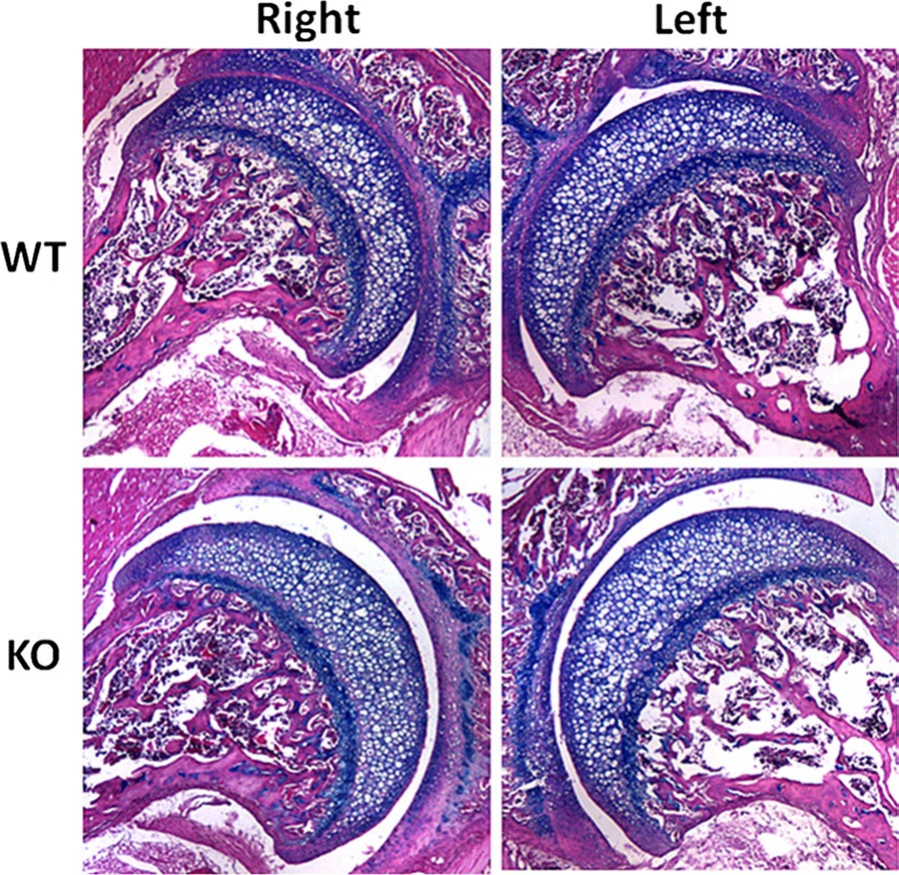
Histology of hip joint of wild type and KO 5 week old mice. Hematoxylin and Eosin stained sections

Investigation of the cellular architecture was performed using histologic examination of the acetabula. Normal cellular morphology of the articular cartilage and subchondral bone were observed in the KO and WT mice with no discernable differences between the right and left side (Fig. 6). Interestingly, we did notice a larger gap between the acetabulum and the femoral head in most of the KO mice sections compared to the WT mice. Unfortunately, before sectioning it was not possible to precisely control the plane of sectioning and thus the significance of the observed gap is unclear. Further work will be necessary to determine if this is real or artifact. Immuno-staining for green fluorescent protein expressed in our KO mice in place of CX3CR1 revealed gene expression in bone marrow cells as has been previously described [13].

A limitation of this study is that we have not shown that mice with this chemokine ablated are more prone to develop hip OA. This point will be addressed in further longer term studies. Additionally, for reasons of cost the rs3732378 variant, found strongly linked to DDH in a large affected family, was not knocked into mice. It is noteworthy however that the presence of this variant is predicted to have function-inhibiting effects on this receptor and has been associated with pathology in other organ systems [10].

## Conclusion

Despite the aforementioned limitations, the described mouse model can provide insight into gene function and its influence on the development of the hip in general and the acetabulum in particular. The effect of CX3CR1 deletion on murine acetabular development in this study provides suggestive evidence of a susceptibility inducing role of the rs3732378 variant in the DNA co-inherited by all 11 affected members of a large family and strongly correlated with DDH in the Chinese population. Further analysis of animal models with this and other potential susceptibility inducing mutations should provide insight into the mechanism of hip development and the pathoetiology of DDH.

## Additional files

**Additional file 1: Figure S1.** 95% Confidence Interval of KO-WT difference in normalized left hip socket diameter as a function of slice number for 5 week old mice. Green line is upper limit, blue line is average, red line is lower limit. Right hip showed no significant diameter differences between KO and control.

**Additional file 2: Figure S2.** All 5 weeks old mouse hip socket diameter measurements.

**Additional file 3: Figure S3.** All 8 weeks old mouse hip socket diameter measurements.

## Abbreviations

OA: osteoarthritis
KO: knock out
WT: wild type
H and E: hematoxylin and eosin
BV/TV: bone volume divided by total volume

## References

[1] Jacobsen S, Sonne-Holm S, Søballe K, Gebuhr P, Lund B (2005). Hip dysplasia and osteoarthritis: a survey of 4,151 subjects from the Osteoarthrosis Substudy of the Copenhagen City Heart Study. Acta Orthop.

[2] Rubini M, Cavallaro A, Calzolari E, Bighetti G, Sollazzo V (2008). Exclusion of COL2A1 and VDR as developmental dysplasia of the hip genes. Clin Orthop Relat Res.

[3] Stein-Zamir C, Volovik I, Rishpon S, Sabi R (2008). Developmental dysplasia of the hip: risk markers, clinical screening and outcome. Pediatr Int.

[4] Wilkinson JA (1992). Etiologic factors in congenital displacement of the hip and myelodysplasia. Clin Orthop Relat Res.

[5] Coleman SS (1968). Congenital dysplasia of the hip in the Navajo infant. Clin Orthop.

[6] Wynne-Davies R (1970). Acetabular dysplasia and familial joint laxity: two etiological factors in congenital dislocation of the hip. A review of 589 patients and their families. J Bone Joint Surg Br.

[7] Edelstein J (1966). Congenital dislocation of the hip in the Bantu. J Bone Joint Surg Br.

[8] Stevenson DA, Mineau G, Kerber RA, Viskochil DA, Schaefer C, Roach JW (2009). Familial predisposition to developmental dysplasia of the hip. J Pediatr Orthop.

[9] Weinstein SL (1987). Natural history of congenital hip dislocation (CDH) and hip dysplasia. Clin Orthop Relat Res.

[10] Feldman GJ, Parvizi J, Levenstien M, Scott K, Erickson JA, Fortina P, Devoto M, Peters CL (2013). Developmental dysplasia of the hip: linkage mapping and whole exome sequencing identify a shared variant in CX3CR1 in all affected members of a large multigeneration family. J Bone Miner Res.

[11] Li L, Wang X, Wang BB (2016). et al CX3CR1 polymorphisms associated with increased risk of developmental dysplasia of the hip in human. J Orthop Res.

[12] Hampton TG, Stasko MR, Kale A, Amende I, Costa AC (2004). Gait dynamics in trisomic mice: quantitative neurological traits of Down syndrome. Physiol Behav..

[13] Zerbini P, Bonifacio E, Piemonti L (2005). Bone marrow mesenchymal stem cells express a restricted set of functionally active chemokine receptors capable of promoting migration to pancreatic islets. Blood.

[14] http://orthoinfo.aaos.org/topic.cfm?topic=a00347.

[15] Constantinou M, Barrett R, Brown M, Mills P (2014). Spatial-temporal gait characteristics in individuals with hip osteoarthritis: a systematic literature review and meta-analysis. J Ortho Sports Phys Ther.

[16] Brach JS, Perera S, Verghese J (2010). Meaningful change in measures of gait variability in older adults. Gait Posture.

[17] Poulet B, de Sousa R, Pitsillides AA (2014). Modifications of gait as predictors of natural osteoarthritis progression in STR/Ort mice arthritis. Rheumatology.

[18] Parvathy SS, Masocha W (2013). Gait analysis of C57BL/6 mice with complete Freund’s adjuvant-induced arthritis using the CatWalk system. BMC Musculoskelet Disord.

[19] Gadalla KKE, Ross PD, Riddell JS, Bailey MES, Cobb SR (2014). Gait analysis in a Mecp2 knockout mouse model of rett syndrome reveals early-onset and progressive motor deficits. PLoS ONE.

[20] Hoshino A, Ueha S, Hanada S, Imai T, Ito M, Yamamoto K, Matsushima K, Yamaguchi A, Iimura T (2013). Roles of chemokine receptor CX3CR1 in maintaining murine bone homeostasis through the regulation of both osteoblasts and osteoclasts. J Cell Sci.

