# Supplementary figures and images for: A murine model for developmental dysplasia of the hip: ablation of CX3CR1 affects acetabular morphology and gait
Source: J Transl Med. 2017 Nov 10;15:233. doi: 10.1186/s12967-017-1335-0 (PMC5681830; doi:10.1186/s12967-017-1335-0)

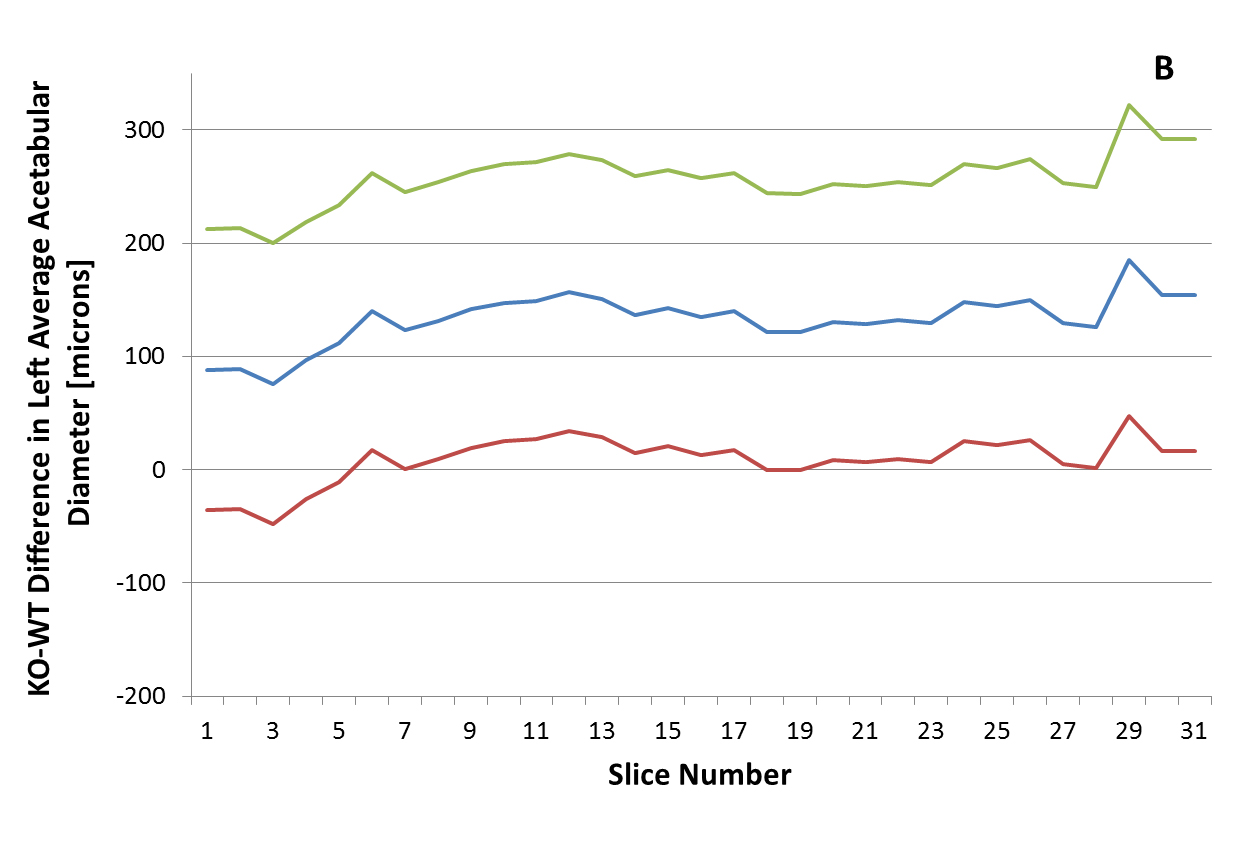

Supplement: Supplementary file 1 — Additional file 1: Figure S1. 95% Confidence Interval of KO-WT difference in normalized left hip socket diameter as a function of slice number for 5 week old mice. Green line is upper limit, blue line is average, red line is lower limit. Right hip showed no significant diameter differences between KO and control. [file 12967_2017_1335_MOESM1_ESM.jpg]
